# Big data-based risk assessment of poultry farms during the 2020/2021 highly pathogenic avian influenza epidemic in Korea

**DOI:** 10.1371/journal.pone.0269311

**Published:** 2022-06-07

**Authors:** Hachung Yoon, Ilseob Lee, Hyeonjeong Kang, Kyung-Sook Kim, Eunesub Lee

**Affiliations:** Veterinary Epidemiology Division, Animal and Plant Quarantine Agency, Gimcheon, Gyeongsangbuk-do, Republic of Korea; Erasmus University Medical Center, NETHERLANDS

## Abstract

Outbreaks of H5-type highly pathogenic avian influenza (HPAI) in poultry have been reported in various parts of the world. To respond to these continuous threats, numerous surveillance programs have been applied to poultry raising facilities as well as wild birds. In Korea, a surveillance program was developed aimed at providing a preemptive response to possible outbreaks at poultry farms. The purpose of this study is to comprehensively present the risks of HPAI evaluated by this program in relation to actual outbreak farms during the epidemic of 2020/2021. A deep learning-based risk assessment program was trained based on the pattern of livestock vehicles visiting poultry farms and HPAI outbreaks to calculate the risk of HPAI for farms linked by the movement of livestock vehicles (such farms are termed “epidemiologically linked farms”). A total of 7,984 risk assessments were conducted, and the results were categorized into four groups. The proportion of the highest risk level was greater in duck farms (13.6%) than in chicken farms (8.8%). Among the duck farms, the proportion of the highest risk level was much greater in farms where breeder ducks were raised (accounting for 26.4% of the risk) than in farms where ducks were raised to obtain meat (12.8% of the risk). A higher risk level was also found in cases where the species of the outbreak farm and epidemiologically linked farms were the same (proportion of the highest risk level = 13.2%) compared to that when the species between the two farms were different (7.9%). The overall proportion of farms with HPAI outbreaks among epidemiologically linked farms (attack rate, AR) was 1.7% as HPAI was confirmed on 67 of the 3,883 epidemiologically linked farms. The AR was highest for breeder ducks (15.3%) among duck farms and laying hens (4.8%) among chicken farms. The AR of the pairs where livestock vehicles entered the inner farm area was 1.3 times (95% confidence interval: 1.4–2.9) higher than that of all pairs. With the risk information provided, customized preventive measures can be implemented for each epidemiologically linked farm. The use of this risk assessment program would be a good example of information-based surveillance and support decision-making for controlling animal diseases.

## Introduction

Avian influenza is an infection of a virus belonging to the genus *Alphainfluenzavirus* of the family Orthomyxoviridae. Poultry, including chicken (Galliformes) and ducks (Anseriformes), as well as wild ducks, geese, and swans (Anseriformes), and other water birds (Charadriiformes) are highly susceptible [1]. Since 2003, outbreaks of H5-type highly pathogenic avian influenza (HPAI) in poultry have been reported in various parts of the world. To respond to the continuous threats from HPAI, numerous surveillance programs have been applied to poultry and wild birds. These programs focus on the timely detection of the avian influenza virus in wild birds and on accurately recognizing the risk of poultry using an early detection system [2], which could assist animal health authorities and farm managers to respond preemptively to HPAI risks [2, 3]. To date, there has been limited use of such information systems. Recently, there has been increasing use of artificial intelligence, particularly deep learning to support risk-based decisions to counter the spread of infectious diseases [4, 5]. However, there are very few reports of artificial intelligence being applied to manage infectious diseases in livestock [6].

Between November 26, 2020, and April 6, 2021 (a period of 132 days), outbreaks of HPAI subtype H5N8 were confirmed at 109 poultry farms in the Republic of Korea [7]. Epidemiological studies from previous epidemics of HPAI have revealed that the major cause of the virus entering Korea was the seasonal movement of migratory birds, and the introduction of the virus into farms was linked to the contamination of the surrounding environment by wild birds (including migratory birds) as well as the entry of people and vehicles into farms [8]. Taking these factors into consideration, animal health authorities have been implementing stronger biosecurity measures to minimize the damage to the poultry industry resulting from HPAI outbreaks. These include restricting the entry of livestock vehicles into farms, except when absolutely necessary (such as feed supply), and disinfecting vehicles at stations operated by local governments prior to the vehicles entry in livestock facilities [9]. In addition, with the aim of providing information for a preemptive response, a deep learning-based risk assessment program was developed to quantify the risks related to vehicle movement [10]. The purpose of this study is to comprehensively present the risks of HPAI evaluated by this program in relation to actual outbreak farms during the epidemic of 2020/2021 in Korea.

## Materials and methods

### Risk assessment program

The risk assessment program used in this study was developed based on deep learning computation. The program was trained using the pattern of livestock vehicles visiting poultry farms and HPAI outbreaks to calculate the risk of HPAI for farms linked by the movement of livestock vehicles (such farms are termed “epidemiologically linked farms”). Livestock vehicles that visited an outbreak farm at some point within 21 days from the date of outbreak were tracked according to the standard operation procedure for avian influenza of Korea [11], and the outbreak of HPAI on the epidemiologically linked farms was checked. The detailed algorithm of this model is described in a previously published study [12].

The movement of livestock vehicles was tracked using a global positioning satellite (GPS) system. In accordance with Article 17–3 of the Korean Act on the Prevention of Contagious Animal Disease [13], vehicles accessing livestock-related facilities must be registered and must have installed a wireless device to record and transmit information on access to such facilities. A few ranges of geo-coordinates in the shape of circles and polygons have been established to identify what livestock vehicles access. Whether vehicles only access the entrance of a farm or move further and enter the inner area of the farm can be confirmed using the range of geo-coordinates of the farms and the GPS of the vehicles. All relevant information is managed through the Korea Animal Health Integrated System (KAHIS) operated by the Animal and Plant Quarantine Agency (http://kahis.go.kr).

The association among outbreak farms, epidemiologically linked farms, outbreaks at epidemiologically linked farms, and livestock vehicles linking those farms was expressed as four categories of risk: from highest risk, ++++, decreasing to +++, ++, and +. The risk assessment program is available through the KAHIS web page (http://kahis.go.kr), which can only be accessed by authorized personnel because of data privacy protection. During the epidemic of 2020/2021, immediately after a suspected case was notified, the risk of HPAI was evaluated for epidemiologically linked farms. The results of the risk assessment were provided to animal health authorities through the official document system of the Korean government.

### Attack rate and statistical analysis

Attack rate (AR) was estimated by taking the proportion of farms with HPAI outbreak among epidemiologically linked farms. Odds ratio (OR) was estimated to compare two proportions (including ARs). To calculate AR, OR, and the 95% confidence interval (95% CI), an internet-based calculator [14] was used.

## Results

During the HPAI epidemic of 2020/2021 in Korea, the majority (103, 94.5%) of the 109 outbreaks occurred in chickens (55 farms, 50.5%) and ducks (48 farms, 44.0%). The rest of the outbreaks were in quails (3 farms, 2.8%), pet or exotic birds (2 sites, 1.8%), and one farm (0.9%) where various species were raised.

The program assessed risk for 7,984 pairs of outbreak farms and the epidemiologically linked farms. The epidemiologically linked farms of these pairs were 5,727 (71.7%) chickens, 2,087 (26.2%) ducks, and 170 (2.1%) other poultry species. The highest risk level of ++++ was predicted 849 times (10.6%); the next highest level of +++, 494 times (6.2%); ++, 651 times (8.2%); and +, 5,990 times (75.0%). The proportion of the highest risk level of ++++ was larger (OR = 1.6, 95% CI: 1.4–1.9) in duck farms (283 of 2,087, 13.6%) compared to chicken farms (504 of 5,727, 8.8%). Among duck farms, the proportion of the highest risk level was larger (OR = 2.4, 95% CI: 1.6–3.7) in breeders (32 of 123, 26.0%) than in meat ducks (251 of 1,964, 12.8%). AR was highest at the risk level ++ (AR = 6.5%, 95% CI: 4.8–8.6), but there was no significant difference with the ARs of +++ (4.3%, 95% CI: 2.8–6.4) and ++++ (4.7%, 95% CI: 3.5–6.4) as their 95% CIs overlapped. However, the ARs of the higher three risk levels were clearly higher than the AR of the risk level + (2.1%, 95% CI: 1.8–2.5; Table 1).

**Table 1:** Risk level predicted by the risk assessment program and Attack Rate (AR) according to poultry farm type.

Risk level	Ducks			Chickens					Others	Total	HPAI outbreak	
	Breeder ducks	Meat ducks	Ducks subtotal	Breeder chickens	Laying hens	Broiler chickens	Native breeds	Chickens subtotal			Number	AR, %
												(95% CI)
++++	32	251	283	45	217	123	119	504	62	849	40	4.7
	(26.4%)	(12.8%)	(13.6%)	(10.4%)	(12.5%)	(6.7%)	(6.9%)	(8.8%)	(36.5%)	(10.6%)		(3.5–6.4)
+++	16	156	172	22	123	83	86	314	8	494	21	4.3
	(13.2%)	(7.9%)	(8.2%)	(5.1%)	(7.1%)	(4.5%)	(5.0%)	(5.5%)	(4.7%)	(6.2%)		(2.8–6.4)
++	13	198	211	51	151	121	100	423	17	651	42	6.5
	(10.8%)	(10.1%)	(10.1%)	(11.8%)	(8.7%)	(6.6%)	(5.8%)	(7.4%)	(10.0%)	(8.2%)		(4.8–8.6)
+	62	1,359	1,421	313	1,244	1,504	1,425	4,486	83	5,990	128	2.1
	(50.4%)	(69.2%)	(68.1%)	(72.6%)	(71.7%)	(82.2%)	(82.3%)	(78.3%)	(48.8%)	(75.0%)		(1.8–2.5)
Total	123	1,964	2,087	431	1,735	1,831	1,730	5,727	170	7,984	231	2.9
			(26.2)					(71.7%)	(2.1%)			(2.6–3.3)

In cases where the farm type of the outbreak farm and epidemiologically linked farm was the same, the proportion of the highest risk level (++++, 546 of 4,139, 13.2%) was higher (OR = 1.8, 95% CI: 1.5–2.1) than that when the farm type was different (303 of 3,845, 7.9%). When their farm types were identical, the proportion of the highest risk level was larger (OR = 1.2, 95% CI: 1.0–1.4) in ducks (262 of 1,839, 14.2%) than in chickens (274 of 2,263, 12.1%). With respect to duck farms, the farms with breeder ducks accounted for the largest proportion (17 of 53, 32.1%). Similarly, in chickens, farms with laying hens made up the largest portion of highest risk (198 of 1,500, 13.2%). When the farm type was the same (AR = 5.1, 95% CI: 4.4–5.8), the confirmation of HPAI on the epidemiologically linked farm was higher (OR = 6.0, 95% CI: 4.1–8.8) than was the case for farms of different types (AR = 0.8, 95% CI: 0.6–1.2; Table 2).

**Table 2:** Risk level and Attack Rate (AR) according to farm type identicality between outbreak farm and epidemiologically linked farms.

Identicality	Risk level	Farm type of the HPAI outbreak farm										HPAI outbreak	
		Ducks			Chickens					Others	Total		
		Breeder ducks	Meat ducks	Ducks subtotal	Breeder chickens	Laying hens	Broiler chickens	Native breeds	Chickens subtotal			Number	AR, %
													(95% CI)
Same	++++	17	245	262	13	198	62	1	274	10	546	39	7.7
		(32.1%)	(13.7%)	(14.2%)	(10.9%)	(13.2%)	(9.8%)	(8.3%)	(12.1%)	(27.0%)	(13.2%)		(5.6–10.6)
	+++	4	142	146	6	107	45	1	159	3	308	16	5.5
		(7.5%)	(8.0%)	(8.0%)	(5.0%)	(7.1%)	(7.1%)	(8.3%)	(7.0%)	(8.1%)	(7.4%)		(3.3–9.0)
	++	8	169	177	7	125	46	1	179	5	361	39	12.5
		(15.1%)	(9.5%)	(9.5%)	(5.9%)	(8.3%)	(7.3%)	(8.3%)	(7.9%)	(13.5%)	(8.7%)		(9.0–17.4)
	+	24	1,230	1,254	93	1,070	479	9	1,651	19	2,924	105	3.7
		(45.3%)	(68.9%)	(68.9%)	(78.2%)	(71.3%)	(75.8%)	(75.0%)	(73.0%)	(51.4%)	(70.6%)		(3.1–4.5)
	Subtotal	53	1,786	1,839	119	1,500	632	12	2,263	37	4,139	199	5.1
													(4.4–5.8)
Different	++++	47	82	129	9	125	5	14	153	21	303	1	0.3
		(9.1%)	(8.8%)	(8.9%)	(4.7%)	(7.2%)	(6.1%)	(23.0%)	(7.4%)	(6.6%)	(7.9%)		(0.1–1.9)
	+++	27	21	48	15	84	10	3	112	26	186	5	2.8
		(5.2%)	(2.3%)	(3.3%)	(7.9%)	(4.8%)	(12.2%)	(4.9%)	(5.4%)	(8.1%)	(4.8%))		(1.2–6.5)
	++	36	67	103	16	146	1	1	164	23	290	3	1.1
		(7.0%)	(7.2%)	(7.1%)	(8.4%)	(8.4%)	(1.2%)	(1.6%)	(7.9%)	(7.2%)	(7.5%)		(0.4–3.1)
	+	406	759	1,165	151	1,391	66	43	1,651	250	3,066	23	0.8
		(78.7%)	(81.7%)	(80.6%)	(79.1%)	(79.7%)	(80.5%)	(70.5%)	(79.4%)	(78.1%)	(79.7%)		(0.5–1.1)
	Subtotal	516	929	1,445	191	1,746	82	61	2,080	320	3,845	32	0.8
													(0.6–1.2)
Total		123	1,964	2,087	431	1,735	1,831	1,730	5,727	170	7,984	231	2.9
													(2.6–3.3)

For some epidemiologically linked farms, risk assessment was conducted in association with two or more outbreak farms. The lower 50% and 75% of epidemiologically linked farms were associated with one- and two-outbreak farms, respectively. On the other hand, there was a single farm that was epidemiologically linked to 35 outbreak farms. After removing duplications, the number of epidemiologically linked farms was 3,883, the vast majority (3,072, 79.1%) of which were chicken farms, followed by 701 (18.1%) ducks and 110 (2.8%) other species. Out of these epidemiologically linked farms, outbreaks of HPAI were confirmed in 67 farms (1.7%, 95% CI: 1.4–2.2). These were 31 duck farms (46.3%) and 36 chicken farms (53.7%). Species-specific ARs were 4.4% (95% CI: 3.1–6.2) in ducks and 1.2% (95% CI: 0.9–1.6) in chickens, so the AR in ducks is higher (OR = 3.9, 95% CI: 2.4–6.4) than that in chickens. The AR was highest for breeder ducks (15.3%, 95% CI: 8.2–26.5) among duck farms and laying hens (4.8%, 95% CI: 3.5–6.7) among chicken farms (Table 3).

**Table 3:** Farm type identicality between HPAI outbreak farms and epidemiologically linked farm, and the Attack Rates (AR) on epidemiologically linked farms.

Farm type of the epidemiologically linked farms		Identicality with outbreak farms								
		Same			Different			All (no duplication)		
		Number of epidemiologically linked farms	Number of HPAI outbreaks	AR, %	Number of epidemiologically linked farms	Number of HPAI outbreaks	AR, %	Number of epidemiologically linked farms	Number of HPAI outbreaks	AR, %
				(95% CI)			(95% CI)			(95% CI)
Ducks	Breeder ducks	44	9	25.7	70	6	9.4	59	9	15.3
				(12.6–52.7)			(4.2–21.2)			(8.2–26.5)
	Meat ducks	1,067	39	3.8	178	2	1.1	642	22	3.4
				(2.8–5.2)			(0.3–4.2)			(2.3–5.1)
	Ducks subtotal	1,111	48	4.5	248	8	3.3	701	31	4.4
				(3.4–6.0)			(1.7–6.7)			(3.1–6.2)
Chickens	Breeder chickens	83	1	1.2	312	8	2.6	169	2	1.2
				(0.2–7.0)			(1.3–5.2)			(0.3–4.2)
	Laying hens	1,024	66	6.9	235	16	7.3	684	33	4.8
				(5.4–8.8)			(4.4–12.1)			(3.5–6.7)
	Broiler chickens	286	1	0.4	1,199	0	-	907	1	0.1
				(0.1–2.0)						(0–0.6)
	Native breeds	12	-	-	1,718	1	0.1	1,312	0	-
							(0–0.3)			
	Chickens subtotal	1,405	68	5.1	3,464	25	0.7	3,072	36	1.2
				(4.0–6.5)			(0.5–1.1)			(0.9–1.6)
Others		32	0	-	133	0	-	110	0	-
Total		2,548	116	4.8	3,845	33	0.9	3,883	67	1.7
				(4.0–5.7)			(0.6–1.2)			(1.4–2.2)

Among 7,984 pairs of outbreak farms and the epidemiologically linked farms that were linked by livestock vehicles, vehicle types could be identified in 7,873 pairs, excluding only 111 (1.4%). The HPAI was confirmed on the epidemiologically linked farms of the 229 (AR = 2.9%, 95% CI: 2.6–3.3) pairs. The vehicle type-specific AR was highest for the vehicles of livestock breeding facility managers (6 of 58 pairs, AR = 10.3%, 95% CI: 4.8–20.8), followed by veterinary pharmaceutical transport (13 of 241, AR = 5.4%, 95% CI: 3.2–9.0) and egg tray transport (17 of 328, AR = 5.2, 95% CI: 3.3–8.1). The entry of the livestock vehicle in the epidemiologically linked farm could be identified in 5,003 (63.5%) pairs. In 2,919 (58.3%) pairs, the vehicles entered the inner farm areas; HPAI was confirmed in 108 (AR = 3.7%, 95% CI: 3.1–4.5) of these pairs. The AR of the pairs where vehicles entered the inner area of the epidemiologically linked farm was higher (OR = 1.3, 95% CI: 1.0–1.6) than that of all the 7,873 pairs. For the vehicles entering the epidemiologically linked farms, the vehicles of livestock breeding facility managers (2 of 18 pairs, AR = 11.1%, 95% CI: 3.1–32.8) showed the highest AR (Table 4).

**Table 4:** Number of visits by livestock vehicles on the epidemiologically linked farms and Attack Rate (AR) by vehicle type.

Vehicle type	All vehicles			Vehicles accessing the inner area of the epidemiologically linked farms		
	Number of visits by livestock vehicles	Number of visits on epidemiologically linked farms confirming HPAI	AR, %	Number of visits by livestock vehicles	Number of visits on epidemiologically linked farms confirming HPAI	AR, %
			(95% CI)			(95% CI)
Livestock breeding facilities manager	58	6	10.3 (4.8–20.8)	18	2	11.1 (3.1–32.8)
Veterinary pharmaceutical transport	241	13	5.4 (3.2–9.0)	70	5	7.1 (3.1–15.7)
Egg tray transport	328	17	5.2 (3.3–8.1)	154	13	8.4 (5.0–13.9)
Egg transport	456	19	4.2 (2.7–6.4)	168	12	7.1 (4.1–12.1)
Compost transport	149	6	4.0 (1.9–8.5)	60	2	3.3 (0.9–11.4)
Livestock manure transport	107	4	3.7 (1.5–9.2)	48	2	4.2 (1.2–14.0)
Sample collection & quarantine	1,523	54	3.6 (2.7–4.6)	232	17	7.3 (4.6–11.4)
Feed transport	2,562	74	2.9 (2.3–3.6)	1,042	44	4.2 (3.2–5.6)
Husk, bran, sawdust, litter transport	191	4	2.1 (0.8–5.3)	81	4	4.9 (1.9–12.0)
Consulting	301	5	1.7 (0.7–3.8)	54	0	-
Live animals transport	1,467	22	1.5 (1.0–2.3)	871	6	0.7 (0.3–1.5)
Veterinarians, Vaccination personnel	330	4	1.2 (0.5–3.1)	85	1	1.2 (0.2–6.8)
Poultry loading & unloading personnel transport	21	0	-	15	0	-
Others	138	1	0.7 (0.1–4.0)	21	0	-
Total	7,873	229	2.9(2.6–3.3)	2,929	108	3.7(3.1–4.5)

The number of livestock vehicles that visited HPAI outbreak farms and epidemiologically linked farms was 903. Only 93 (10.1%) of these vehicles visited two or more outbreak farms. The vehicle types that visited the largest number of HPAI outbreak farms were feed lorries and egg tray transport, and they visited up to four farms. On the other hand, the number of epidemiologically linked farms visited by one livestock vehicle was four in median (the first quartile = 2; the third quartile = 10). Of these 903 livestock vehicles, 136 (15.1%) visited 67 epidemiologically linked farms where HPAI outbreak was confirmed. Five hundred and thirty-eight (59.6%) livestock vehicles entered the inner areas of the 1,358 epidemiologically linked farms, and HPAI was confirmed in 47 (3.5%, 95% CI: 2.6–4.6) farms visited by 74 (13.8%) vehicles. The AR associated with vehicles entering the epidemiologically linked farms was higher (OR = 2.0, 95% CI: 1.4–3.0) than the overall AR of 1.7% (67 of 3,883 epidemiologically linked farms).

## Discussion

During the 2020/2021 HPAI epidemic in Korea, chicken farms accounted for the highest number (absolute) of both outbreak farms and epidemiologically linked farms. However, in terms of the relative number of outbreaks, duck farms experienced over five times the number of outbreaks than did chicken farms. As of the fourth quarter of 2020, the numbers of farms rearing more than 3,000 chickens and 2,000 ducks were 2,845 and 449, respectively. The proportions of these farms with HPAI were 1.9% for chickens and 10.7% for ducks [15].

In the present study, we found that the proportion of the highest risk level was greater in duck farms than in chicken farms. Moreover, the highest risk was predicted in the type of farms that regularly send out eggs, such as breeder ducks and laying hens (Tables 1 and 2). On the other hand, in epidemiologically linked farms of the same farm type as the outbreak farms, the predicted risk was higher and a greater number of outbreaks were confirmed (Tables 2 and 3). In addition, a larger number of outbreaks were detected in epidemiologically linked farms where vehicles accessed the interior area of the farm (Table 4). In our previous study, a higher risk was found for epidemiologically linked farms confirming HPAI than for those that had not [12]; this fact emphasizes the need of implementing appropriate preemptive measures on high risk farms.

The association between HPAI outbreaks and disease transmission and its relation to the structure of the poultry industry, including production systems and value chain, has been demonstrated through numerous studies in many countries [16–21]. The regular entry of people and vehicles for loading–unloading poultry or shipping-out products (i.e., eggs) and excretions can increase the risk of the virus spreading [22, 23]. Certain types of poultry farms are affiliated with associations or agencies for the seamless supply and availability of vehicles and personnel on a regular schedule; examples are duck producer associations in communities in France [24] and the affiliation of businesses producing ducks or broiler chickens in Korea. The sharing of vehicles and staff is common in these affiliations. In these situations, insufficient operational biosecurity measures increase the risk of HPAI outbreaks [25–27]. Rather than indiscriminately strengthening disease control measures for all farms with linkages to an affiliation, efficient control can be achieved by focusing on implementing biosecurity measures after identifying potential spreader or receiver farms with a high risk [22].

By focusing on the movement of livestock vehicles connecting the HPAI outbreak farms with the epidemiologically linked farms, we have examined whether the HPAI was actually confirmed in farms that were predicted to be at a high risk. The reason for conducting risk assessment in relation to HPAI outbreak in poultry farms was to demonstrate the risk to epidemiologically linked farms and to help in preparing farm managers and animal health officials respond with appropriate actions. It should be clarified that the goal of the study was not to establish a cause–effect relationship between the source and receiver farms by the movement of livestock vehicles. In that sense, the risk assessment was conducted immediately after a suspected case was notified, rather than waiting until the entire epidemiological investigation could be presented.

In some cases, it was not possible to verify whether a visiting livestock vehicle had accessed the inner area of a farm. The establishment of geo-coordinate ranges on farms—to know whether livestock vehicles access the interior of the farm—is gradually expanding to include all livestock farms, starting with breeder ducks and laying hens in the case of poultry, and pigs in the case of cloven-hoofed animals. Although it was confirmed for only a part of all epidemiologically linked farms, a higher risk and more outbreaks were found when livestock vehicles actually accessed the interior part of the farm. Considering this, it may be necessary to implement reinforced control measures, particularly banning livestock vehicles from accessing the interior of poultry farms.

In an emergency brought about by an outbreak of a transboundary animal disease, such as HPAI, readily available information is necessary for both the national and local animal health authorities to deal with time pressures, insufficient human and physical resources, and general uncertainty and to implement the appropriate response [28]. Decision-making support systems had been implemented decades ago for emergency disease control against diseases in swine, such as foot-and-mouth disease or classical swine fever, and measures have been established to comprehensively utilize information-based on the advice of experts and through simulation modeling [29–31]. On the one hand, previous disease outbreaks and the information acquired from them have led to increased public awareness and stronger interest in disease control. In France, an outbreak of 485 cases of H5N8 HPAI was recorded between 2016 and 2017 centered around an area with a high density of duck farms, and it is reported that the experience of such a serious epidemic has led to an increase in awareness of farm owners generally [3]. Nonetheless, there are differences depending on the research. Several reports indicate how the stresses of a disease epidemic have made the farmers implement various disease control measures to prevent an outbreak in their farms [32]; however, there are cases where the stresses have suppressed the new control measures [33]. Anyway, effective communication can promote heightened awareness of the risks and can lead to voluntary action among farmers to implement and regularly practice effective biosecurity measures [3].

## Conclusion

In this study, the risk of HPAI on poultry farms was estimated in relation to the outbreak of HPAI during the epidemic of 2020/2021 in Korea. The Animal and Plant Quarantine Agency provided information on farms at a high risk of HPAI outbreak and vehicles visiting those farms so that animal health authorities could preemptively respond. The evaluated risk can inform the implementation of customized control measures at the site during an epidemic. The main parameters of the current risk assessment program concern the movement of livestock vehicles being tracked with GPS. The parameters used in the program continue to expand and to be complemented. The risk assessment program presented in this study will aid in the development of an information-based decision support system for animal health.
